# Spatial heterogeneity of urban–rural integration and its influencing factors in Shandong province of China

**DOI:** 10.1038/s41598-022-18424-0

**Published:** 2022-08-22

**Authors:** Baoyan Shan, Qiao Zhang, Qixin Ren, Xinwei Yu, Yanqiu Chen

**Affiliations:** School of Surveying and Geo-Informatics, Shandong Jianzhu University, Jinan, 250101 China

**Keywords:** Environmental economics, Socioeconomic scenarios

## Abstract

Based on nighttime light data and statistical data, this study calculated the level of urban–rural integration (URI) of Shandong province, researched spatial heterogeneity of URI levels by local spatial autocorrelation analysis, Geodetector, and geographically weighted regression, and analyzed its influencing factors and spatial heterogeneity. The results concluded that: (1) The spatial pattern of urban–rural integrated level is consistent with the level of regional economic development in Shandong province. The level of URI is higher along the Qingdao–Jinan railway and along the coast, whereas the level is lower in southwest Shandong and northwest Shandong. (2) The cities of Yantai and Weifang are High–High cluster areas of urban integration, and Jining is a Low–Low cluster area. The spatial agglomeration characteristics are not significant in other cities. (3) Among the main factors affecting URI, the explanatory power of the rural population with high school or technical secondary school education or above, the area of urban construction land, and the secondary and tertiary industry GDP to the spatial pattern of URI in Shandong province are 73.58%, 62.08%, and 58.66%, respectively. As the key factors, spatial heterogeneity, such as north–south differences, southwest-to-northeast differences, and east–west differences, is evident.

## Introduction

Urban–rural integration (URI), as spatial patterns realizing urban and rural integration and coordinated development, aims to achieve the coordinated development of urban–rural areas in society, economy, culture, and others^1^. It is a vital path to eliminate urban and rural dual structures, shape new urban–rural relationships, achieve rural revitalization, and advance new urbanization, with building a well-off society being an equally important measure^1,2^. Urban–rural coordination can enable the relationship of economy and social development between cities and countryside to be fundamentally reconstructed, which is crucial for regional sustainable development^3^ and for a coordinated human society^4^. In the era of globalization, agricultural modernization and rural sustainable development are key^5,6^. Therefore, URI has become a significant area of study warranting considerable attention of scholars both at home and abroad. Both developed and developing countries actively explore responses suitable for national conditions of URI and expected results have been achieved^7^.

There are three important aspects in the study of URI: spatial patterns, influencing factors, and promoting measures. Although the regional systems between cities and countryside intersect, fuse, and integrate^8^, the countryside regional function and urban–rural development coordination have clear spatial differences^9,10^. Many factors influence URI, for example, long-standing urban and rural binary structure in China^11,12^, the mass exodus of the rural population because of urbanization process^13^, capital market transfer and institutional arrangement, and price distortion of land market^14^, land finance growth and urban bias, etc.^15^, enlarging the differences between cities and countryside and restricting URI. Rural hollowing out, agricultural marginalization, and the aging of farmers are also important factors restricting the development of URI^16,17^. China proposed rural revitalization strategies responding to the problems in town and country development. Population, land, and industry are the key elements influencing rural social economy development^18^, in which the rural industry is an essential foundation for promoting the integration between cities and countryside^19^, and the advantages and drawbacks of industrial structure will affect the sustainability of socio-economic development of rural areas^20^. In addition, economic agglomeration^21^, market integration^22^, optimal allocation and highly efficient utilization of population^20^, and coordinating countryside regional function and order transformation^23^ all have a positive impact on accelerating URI.

Constructing an index system is the basis for study of the URI level, based mainly on population integration, economic integration, social integration, spatial fusion, life integration, ecological integration, and other aspects^24–29^. Per capita income and consumption of residents between cities and countryside are used sometimes to calculate^2^. Relevant scholars used 12 indicators to calculate the urban–rural integration index of Beijing, with the maximum, minimum and average values of 0.7325, 0.5324 and 0.6158 respectively^30^. There are many research methods, which are relatively mature on the spatial pattern and influential factors of the level of URI. The research methods of spatial patterns are hotspot analysis and global and local Moran’s I index^25,28,29^, and the research methods of influential factors are Geodetector, correlation analysis, regression analysis, and spatial econometric ^21,24,27,29,31^. The nighttime light data is so strongly associated with urban built-up areas and economic indices of a region that researchers applied it to the URI area^32,33^. The index system, constructed by statistical indicators, is comprehensive in the urban–rural integrated level. However, it has a certain level of subjectivity choosing index. Relatively, the nighttime light data is more objective and not conditioned by administrative boundaries, but it is less comprehensive than the index system. This paper integrates statistical indicators and nighttime light data to study the URI level. Firstly, the urban–rural integration level is calculated by using the total brightness of nighttime light data in urban areas, URI areas and rural areas. At the same time, the URI level is calculated by using statistical data, and then the comprehensive level of URI is calculated by using the weights determined by entropy value method, and the spatial pattern of URI level is studied by using GIS. And the main factors influencing URI based on three aspects central city influence, rural development level, and rural–urban connection are analyzed and spatial heterogeneity of its influence is explored.

## Data sources and methodologies

### Study area and data sources

Shandong is a coastal province and a national comprehensive experimental area for the conversion of old and new kinetic energy. It is in a critical period of eliminating backward production capacity, promoting emerging industries, changing development mode, and optimizing economic structure. At the end of 2020, Shandong province had a resident population of 10,047.24 million, a regional GDP of 73,129 billion, and a tertiary industrial structure of 7.3:39.1:53.6. Shandong Province has three core cities, Jinan, Qingdao and Yantai, and 13 districted cities. Among them, the value-added cities of Jinan, Qingdao and Yantai are of secondary industry accounted for 6.7%, 7.9% and 10.7% of the province respectively, and the value-added of tertiary industry of these three cities accounted for 16.0%, 19.4% and 10.3% of the province respectively. The disposable income per capita reached 43,726 yuan, and the per capita consumption reached 27,291 yuan for city residents. For countryside residents, the disposable income per capita reached 18,753 yuan, and the per capita consumption reached 12,660 yuan^34^.

This study derived statistical data from the third agricultural census data of Shandong province and the 2018 Shandong Statistical Yearbook. The vector data map was taken from Tianditu·Shandong (http://www.sdmap.gov.cn/), and NPP/VIIRS remote sensing image data in December 2017 was derived from http://www.ngdc.noaa.gov/. The unit of research was the administrative district in late 2018, including 17 district cities, of which Laiwu city was absorbed into Jinan in 2019.

### Methodologies

#### Range standardization

Due to the different brightness values of nighttime light, statistical indicators, and dimensions of the calculation results, this study range standardized related data and results according to Formula 1 for comparative analysis^35^.

1 $$x_{ij} = \frac{{x_{ij} - \mathop {\min }\limits_{i} \left\{ {x_{ij} } \right\}}}{{\mathop {\max }\limits_{i} \left\{ {x_{ij} } \right\} - \mathop {\min }\limits_{i} \left\{ {x_{ij} } \right\}}}\;\left( {{\text{i}} = {1},{2},{3}...,{\text{ m}};{\text{ j}} = {1},{2},{3 }...,{\text{ n}}} \right)$$

#### Entropy weight method

We chose the entropy weight method to determine the weights of indicators. First, Formula 2, defined as p_ij_, can help calculate the specific gravity of the ith indicator. Then, Formula 3, defined as e_ij_ helps calculate the entropy values for the ith indicator. Finally, we employ Formula 2 to calculate the weight of the i th indicator defined as w_ij_^36^.

2 $$p_{ij} = \frac{{x^{\prime}_{ij} }}{{\mathop \sum \nolimits_{j = 1}^{n} x^{\prime}_{ij} }}$$

3 $$e_{ij} = \frac{{ - \mathop \sum \nolimits_{j = 1}^{n} p_{ij} \cdot \ln p_{ij} }}{\ln n}$$

4 $$w_{ij} = \frac{{1 - e_{ij} }}{{\mathop \sum \nolimits_{i = 1}^{m} \left( {1 - e_{ij} } \right)}}$$

#### Local spatial autocorrelation analysis

Local Moran’s I is a common analytical method for spatial autocorrelation, which expresses the spatial agglomeration characteristics of the local range. This paper applies this method to study the spatial pattern and spatial heterogeneity of the urban–rural integration level of each city in Shandong Province, calculated as shown in Formula 5^35^:

5 $$I_{i} = \frac{{\left( {x_{i} - \overline{x}} \right)}}{{s^{2} }}\mathop \sum \limits_{j} w_{ij} \left( {x_{j} - \overline{x}} \right)$$

The significance of the Local Moran’s I is tested using standardized statistic Z, which is calculated according to Formula 6^35^:

6 $${\text{z}}\left( {I_{i} } \right) = \frac{{{\text{I}}_{{\text{i}}} - {\text{E}}\left( {{\text{I}}_{{\text{i}}} } \right)}}{{\sqrt {{\text{VAR}}\left( {{\text{I}}_{{\text{i}}} } \right)} }}$$

In Formula 5, x_i_ and x_j_ are the index values of the study area, representing the composite score of URI development levels of i and j cities in this paper. ®x is the mean of x. Spatial weight matrix, defined as W_ij_, indicates proximity relation of regions i and j, and it is constructed using Queen in this paper. When regions i and j have common sides of the polygons or common points, that is adjacent. In Formula 6, E(I) is the expected value of math and, theoretically, Var(I) is the variance.

#### Geodetector

Geodetector, as a tool, detects spatial heterogeneity, which comprises four parts: the differentiation and factor detector, interaction detector, risk detector, and ecological detector^37,38^. Of these, the differentiation and factor detector, measured by q values, is to explore spatial heterogeneity of the dependent variable Y and the extent to which the independent variables X explain the dependent variable Y. The larger the values of q, the more obvious the spatial heterogeneity of Y. If the independent variables X generate the stratification, larger values of q would indicate that the explanation of the independent variables X on the dependent variable Y is stronger. Conversely, the smaller the q values, the weaker the explanation. According to Formula 7, q values were calculated^38^. Interaction detectors predominantly identify interactions between the independent variables. It can evaluate whether the interaction of two independent variables, such as X1 and X2, will significantly enhance or weaken the explanation of the dependent variable Y or whether these two independent variables affect Y independently of each other. This paper uses Geodetector to study the key factors and their interactions that affect the level of urban–rural integration in Shandong Province.

7 $${\text{q}} = 1 - \frac{{\mathop \sum \nolimits_{h = 1}^{L} N_{h} \partial_{h}^{2} }}{{N\partial^{2} }}$$

In Formula 7, h is the stratification of independent variables X or dependent variable Y. Both ${N}_{h}$ and N are the number of units, the former is for layer h and the latter is for the whole area; ${{\partial }_{h}}^{2}$ and ${\partial }^{2}$ are the variances of layer h and the Y value of the whole region, respectively.

#### Geographically weighted regression

The geographically weighted regression (GWR) method is an extension to the global regression model with geospatial space of data to regression parameters. Formula 8 shows the model^36^. This paper uses GWR to study the spatial heterogeneity of factors affecting the level of urban–rural integration in Shandong Province.

8 $$y_{i} = \beta_{0} \left( {u_{i} ,v_{i} } \right) + \mathop \sum \limits_{j = 1}^{k} \beta_{j} \left( {u_{i} ,v_{i} } \right)x_{ij} + \varepsilon_{i}$$

In Formula 8, β_0_ and β_j_ refers to the coefficients to be determined. ε_i_ is a random error of the ith area, meeting the assumptions of zero mean, homoscedasticity, and independence.

#### Calculation of urban–rural integration level

**Figure 1:**
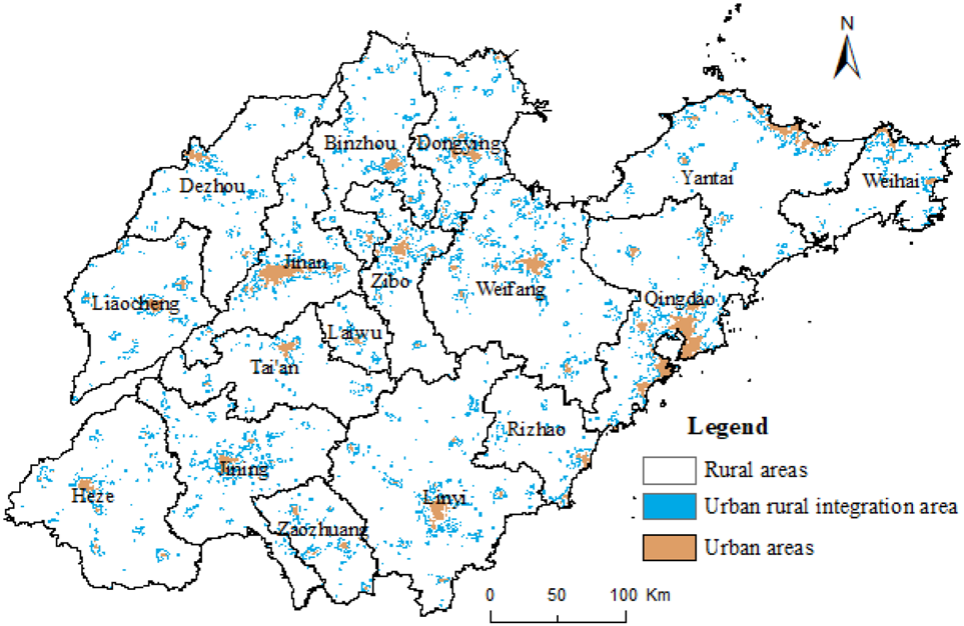
Urban and rural areas of Shandong province based on nighttime light data. This figure was created with ArcGIS 10.5 (URL: http://www.esri.com/).

The mean value of the study area NPP/VIIRS nighttime light brightness is 1.8009, and maximum and minimum values are 466.99 and – 0.02, and the standard deviation of the value is 4.9815. As provided in previous studies^32,33^, with the NPP/VIIRS nighttime light data, the urban–rural level measurement is based on the assumption as follows: There is the largest lighting brightness for the urban built-up area, followed by URI areas and rural areas. Area and total brightness of the urban built-up area reflect urban scaling and development level. The URI area, with large luminance fluctuation, is the critical region reflecting the level of the URI area. The average brightness of the rural area reflects the level of development as well as the URI level. Research ideas and methods are as follows: (1) Thresholds of nighttime light brightness are determined according to the principle that error sum of squares is smallest between the exacted area of nighttime light data and statistics of urban built-up area. When the nighttime light brightness is greater than 8.9, the sum of squares of the error between the extracted area of nighttime light data and the actual area is minimized, so the grid with the value of nighttime light brightness is greater than 8.9, considered as the urban built-up area, and we extracted URI. When the brightness value of nighttime light of the grid is less than or equal to 8.9, we calculate the range of nighttime light brightness using focus statistics of 3 × 3 neighborhood with ArcGIS10.5. Finally, we define the grid with a range of brightness regions greater than or equal to 3.55 as URI area. Others are rural areas. Figure 1 shows the result. (2) First, we calculate the URI areas of each city of Shandong province and nighttime light total luminance with ArcGIS10.5 "show zoning statistics" tool. From this, we calculate the proportion of URI areas of each city to total areas of this city and nighttime light total luminance per unit area of URI area of each city and normalize them separately using Formula 1. The proportion of URI areas of each city to total areas of this city represents coverage of the URI area of each city. While nighttime light total luminance per unit area reflects the development level of URI areas, they collectively reflect URI level of this city. Finally, the expert evaluation method determines the weighting of the two, both equaling 0.5. Accordingly, we calculate the development level of the URI area of each city in Shandong province. (3) Using the same method, we calculate the nighttime light total luminance per unit area in rural of each city, normalized with Formula 1 to reflect the development level in rural areas. (4) Weighting of the level of development of URI areas and rural areas, determined by the expert evaluation method, are 0.7 and 0.3, respectively. Accordingly, we calculate the level of URI of each city in Shandong.

We see URI at a smaller gap between per capita consumption and income in cities and countryside^2,39^. A significant manifestation of URI is that labor productivity and labor income in cities and countryside converge, and there are two significant aspects for URI development, including income gap and consumption level in cities and countryside^2^. Following the previous study^2^, we calculate the urban–rural integrated level using statistics of per capita income and consumption of residents between cities and countryside. The ratio of the difference between income and expenditure to income of urban and rural residents is used to calculate the level of URI development in this literature. However, to reflect the difference of urban–rural integrated levels led to by different incomes, this paper uses the standardized income of residents between cities and countryside after weighting to improve. With the same ratio of the differences between income and expenditure to income of residents between cities and countryside, if the income of residents between cities and countryside is high, the level of URI is considered relatively high, while in smaller ratio, lower incomes have lower urban–rural integrated levels, and the opposite happens when income is higher. The calculation of urban–rural integrated level refers to Formula 9.

9 $${\text{UR}}_{j} = \frac{{\left( {{\text{RE}}_{j} - {\text{RC}}_{j} } \right)/{\text{RE}}_{j} }}{{\left( {{\text{UE}}_{j} - {\text{UC}}_{j} } \right)/{\text{UE}}_{j} }},\;\frac{{{\text{RE}}_{j} }}{{{\text{Max}}\left( {{\text{RE}}_{j} } \right)}},\;\frac{{{\text{UE}}_{j} }}{{{\text{Max}}\left( {{\text{UE}}_{j} } \right)}}$$

where UR_j_ is the index of urban–rural integrated level in j city, and the larger the UR_j_ , the higher will be the level of URI. RE_j_ and RC_j_ represent disposable income per capita and consumption per capita of rural residents in j city, respectively, while UE_j_ and UC_j_ represent per capita disposable income and per capita consumption of urban residents of j city, respectively. Max(UE_j_) and Max(UC_j_) are the maximum of UE_j_ and UC_j_ . Formula 9 calculates the index of the urban–rural integrated level of each city in Shandong province, and Formula 1 standardizes results.

## Study results

### Spatial pattern of urban–rural integration levels in Shandong province

#### Urban–rural integration levels and spatial distribution in Shandong province

**Figure 2:**
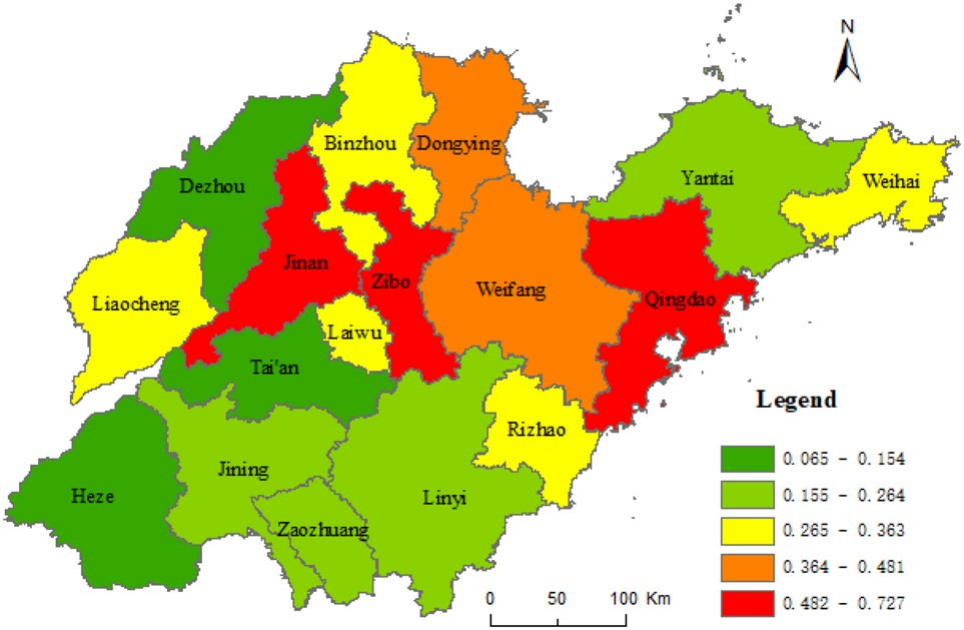
Urban–rural integrated level of Shandong province based on nighttime light data. This figure was created with ArcGIS 10.5 (URL: http://www.esri.com/).

**Figure 3:**
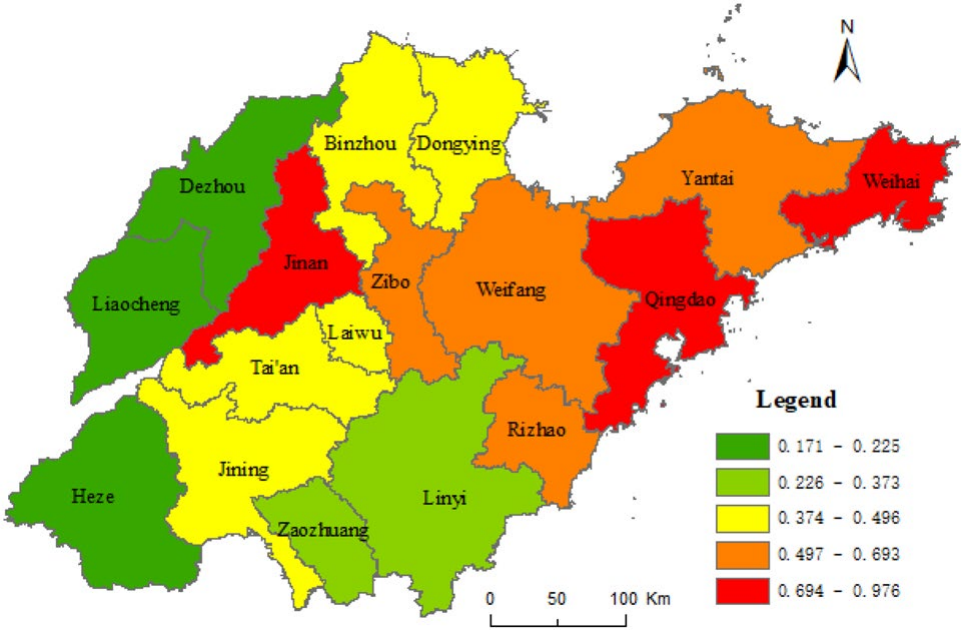
Urban–rural integrated level of Shandong province based on statistical data. This figure was created with ArcGIS 10.5 (URL: http://www.esri.com/).

**Figure 4:**
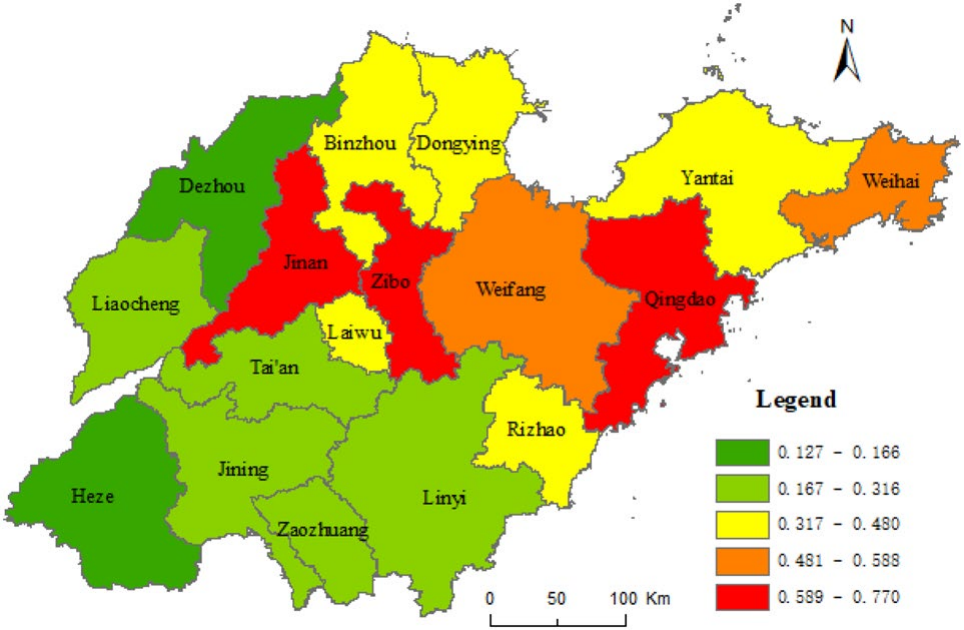
Urban–rural integrated level of Shandong province. This figure was created with ArcGIS 10.5 (URL:http://www.esri.com/).

The calculation results based on nighttime light data and statistics are shown in Table 1. We perform the spatial distribution analysis of the results, considered in five categories by the natural breaks method. Figures 2 and 3 shows the final results. The weight of the two determined by the entropy weight method (Formula 2–4) are 0.5682 and 0.4318, respectively. From this, the results of the urban–rural integrated level in Shandong province are calculated, as shown in Table 1. Figure 4 shows the results divided into five categories using the natural breaks method.

**Table 1:** Results of urban–rural integrated level of cities in Shandong province.

City	The level of urban–rural integration based on nighttime light data	The level of urban–rural integration based on statistical data	The level of urban–rural integration
Qingdao	0.638	0.944	0.770
Jinan	0.571	0.938	0.729
Zibo	0.727	0.588	0.667
Weihai	0.292	0.976	0.588
Weifang	0.436	0.664	0.534
Dongying	0.481	0.479	0.480
Yantai	0.264	0.693	0.449
Rizhao	0.295	0.599	0.426
Binzhou	0.363	0.496	0.421
Laiwu	0.323	0.430	0.369
Jining	0.210	0.456	0.316
Zaozhuang	0.237	0.373	0.296
Liaocheng	0.337	0.225	0.288
Linyi	0.216	0.317	0.260
Taian	0.065	0.489	0.248
Dezhou	0.154	0.182	0.166
Heze	0.094	0.171	0.127

There are significant differences in the level of URI of each city in Shandong as shown in Table 1 and Figs. 2 and 3, calculated based on nighttime light data and statistical data. Comparison between the calculated results based on statistical data and those based on nighttime light data, Qingdao, Jinan and Weifang are in the same order; Zaozhuang, Linyi, Dezhou and Heze City are 1 bit lower; Binzhou, Laiwu, Zibo, Dongying and Liaocheng are 2–8 bits lower; Weihai, Tai'an, Yantai, Rizhao and Jining are 3–9 bits higher. However, the ranking of the two calculation results has the biggest gap in Weihai, Taian, Yantai, and Liaocheng with the difference in ranking between 7 and 9.

As shown in Table 1 and Fig. 4, with the highest level of URI in Qingdao, Jinan, and Zibo in Shandong, Qingdao and Jinan are the binucleated cities, which are more influential, and their driving effect on the peripheral rural areas is strong; Zibo is a group city with urban–rural interlaced distribution, and it is favorable for driving the surrounding rural areas to achieve development. The higher level of URI is in Weihai and Weifang, of which, Weihai has the higher level of economic development, a relatively small area of city, and a higher economic development level than the cities and countries in Shandong and is beneficial to promote the integration of URI. The cities with the higher and highest levels of URI are along the Qingdao–Jinan railway and the coast. They are regions with higher economic development levels in Shandong. The urban–rural integrated level in Dongying, Yantai, Rizhao, Binzhou, and Laiwu are medium levels; Jining, Zaozhuang, Liaocheng, Taian, and Linyi are at lower levels; and the lowest levels are in Dezhou and Heze. The cities with the lower and lowest levels of URI are in Southwest Shandong and Northwestern Shandon, which are regions with a relative lag of economic development levels in Shandong.

#### Spatial heterogeneity of urban–rural integrated level in Shandong

**Figure 5:**
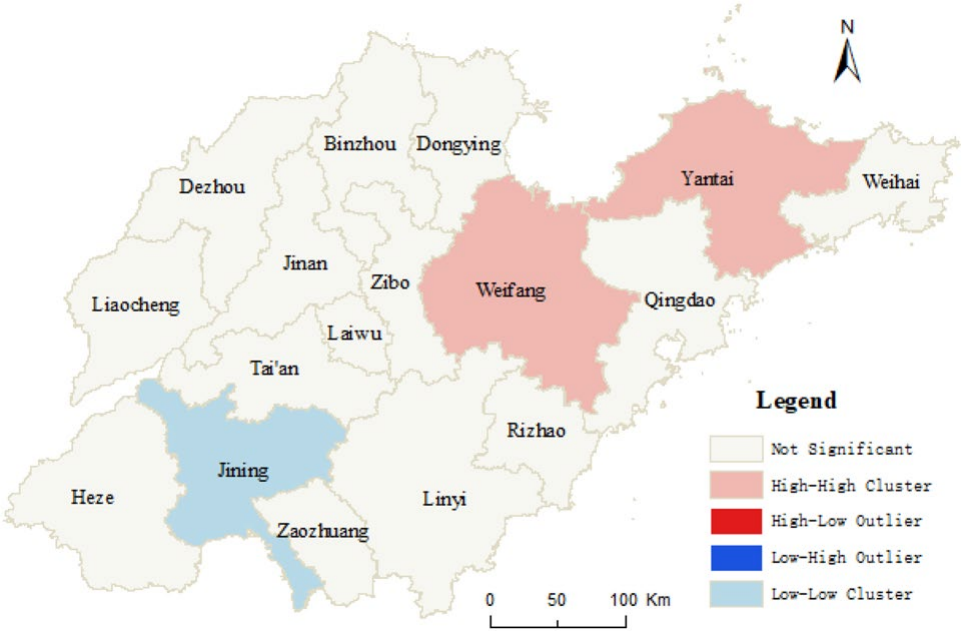
Spatial distribution of hot and cold spots of urban–rural integrated levels in Shandong province. High–High clustering (Low–Low clustering) indicates that the URI level of this city is high (low), and the URL level of its surrounding cities is also high (low); High–Low outlier (Low–High outlier) indicates that the URI level of this city is high (low), but the URL level of its surrounding cities is low (high); Not significant indicates that the local spatial autocorrelation of URI level is not significant. This figure was created with ArcGIS 10.5 (URL: http://www.esri.com/).

The local Moran’s I index, Z score, and *p* value of the comprehensive index of URI level of Shandong cities are calculated with ArcGIS10.5 using Formulas 5 and 6. Table 2 displays the result, and Fig. 5 illustrates the spatial pattern.

**Table 2:** Results of hot-spot analysis of urban–rural integrated level of the cities in Shandong province.

City	Moran’s I	Z score	P value	Type
Jinan	3.12 × 10^–5^	0.5692	0.312	
Qingdao	1.43 × 10^–5^	0.8153	0.226	
Zibo	2.62 × 10^–5^	1.0054	0.182	
Zaozhuang	1.06 × 10^–5^	1.1063	0.150	
Dongying	1.90 × 10^–8^	0.0746	0.466	
Yantai	4.05 × 10^–6^	2.1786	0.022	HH
Weifang	2.04 × 10^–5^	2.0433	0.026	HH
Jining	1.97 × 10^–5^	1.9461	0.022	LL
Taian	7.75 × 10^–6^	0.2532	0.400	
Weihai	1.31 × 10^–6^	0.2032	0.372	
Rizhao	1.55 × 10^–7^	0.2747	0.434	
Laiwu	8.99 × 10^–6^	1.3344	0.102	
Linyi	5.65 × 10^–6^	0.4885	0.322	
Dezhou	2.33 × 10^–5^	0.9265	0.216	
Liaocheng	8.33 × 10^–6^	1.4263	0.122	
Binzhou	8.11 × 10^–8^	0.9826	0.172	
Heze	8.55 × 10^–6^	0.5976	0.422

As shown in Table 2 and Fig. 5, the spatial distribution pattern of the index of the level of URI in Shandong was obvious. Yantai and Weifang were the High–High cluster areas, with the surrounding area, whose level of URI was higher than the provincial average. Jining, as the Low–Low cluster area, indicated that the level of URI in Jining and its surrounding four cities was lower than the provincial average. However, regions with High–Low outliers and Low–High outliers were not detected. The local spatial autocorrelation of urban–rural integrated levels in other cities was not significant, nor was the spatial agglomeration of urban–rural integrated levels.

### Influencing factors and spatial heterogeneity of urban–rural integrated levels in Shandong

#### Factors influencing urban–rural integration

**Figure 6:**
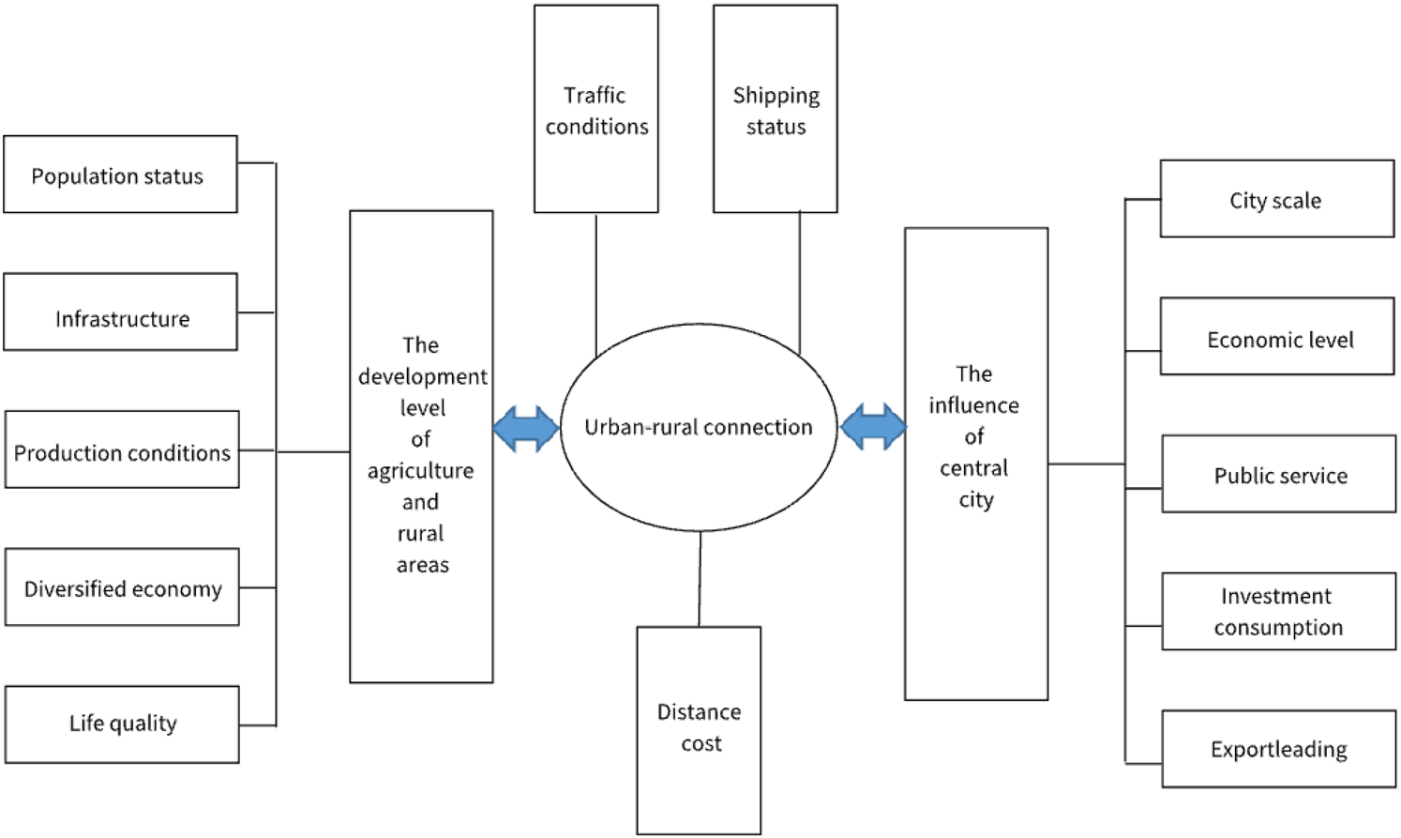
Influencing factors of urban–rural integration.

Urban and rural areas are complex territorial systems with spatial intersection, complementation constructs, and interactions. The urban–rural relations reflect a basic relationship of the dual socio-economic structure of the city and the countryside. Therefore, it is critical for the sustainable development of the region when urban and rural areas integrate and coordinate development. Not only does the level of development of the country itself determine the level of URI, but the influence of the central city also should be considered. Meanwhile, it is associated with the convenience of the connection between cities and countryside. Figure 6 shows their relationship.

The level of development of the country itself includes the status of practitioners, current situation of infrastructure in the rural areas, agricultural production, developing diversified economy in rural areas, the per capita income and consumption of farmers, the life quality of farmers, and so on. The effect of the central city on rural areas depends on size of the cities, economic development level, the situation of infrastructure, as well as investment, consumption, and export. The convenience of the urban–rural relation influences URI, depending upon transport and urban–rural distance. So, Table 3 represents the chosen indicators influencing the level of URI in Shandong.

**Table 3:** Influence index of urban–rural integrated level of Shandong province.

First-level Indices	Second-level Indices	Third-level Indices	Variable
The influence of central city	Economic level	Secondary and tertiary industries GDP	X_1_
		Fixed assets investment	X_2_
		Total export–import volume	X_3_
	City scale	Urban population	X_4_
		Urban construction land area	X_5_
	Public service	The number of health institutions	X_6_
		The number of secondary schools	X_7_
Rural and agricultural development level	Population status	The proportion of high school and secondary school and above	X_8_
		The proportion of population in non-agricultural industry	X_9_
		The proportion of population aged 19–59 years	X_10_
	Infrastructure	The proportion of village that domestic sewage is treated centralized or partially centralized	X_11_
		The proportion of village that have kindergartens and primary schools	X_12_
		The proportion of village that have clinics	X_13_
	Production Situation	The number of tillage equipment	X_14_
		The proportion of agricultural operators that participate in new business organization	X_15_
		The proportion of large-scale agricultural operators and agricultural business units who sell agricultural products by E-commerce	X_16_
	Life quality	The proportion of farmers who using purified tap water as a source of drinking	X_17_
		The proportion of farmers who live mainly on gas, natural gas, and liquefied petroleum gas	X_18_
		Car ownership	X_19_
Urban–rural connection	Traffic condition	Road density	X_20_
		The proportion of village which have entrance and exit of highway	X_21_
		The proportion of village that have railway stations	X_22_
	Transport Capacity	Freight volume	X_23_
		Passenger volume	X_24_

#### Geographical detection of influencing factors of urban–rural integration in Shandong

Geodetector prepared by Excel can help calculate the detection of influencing factors of URI in Shandong province, freely downloaded from http://www.GeoDetector.org^38^. The dependent variable is the URI level index which is the numerical quantities, and the URI impact index is used as the independent variable. Because raw data are all numerical quantities, K-means clustering or the quantile method is used to change the type quantity^37^. In this paper, study areas were classified into five categories by the quantile method with the characteristics of each numeric independent variable: top 20%, (20% 40%], (40% 60%], (60% 80%], (80% 100%]. Due to a large number of independent variables, the relative matrix calculated by the software was larger, such as the interaction detector is 24 × 24 Matrix. We summarize the calculation results, Table 4 shows the factor detection results, and Table 5 shows the interaction detection results.

**Table 4:** Results of factor detector.

Independent variable	q value	p value	Independent variable	*q* value	*p* value
X_1_	0.5866	0.5740	X_13_	0.0253	0.9937
X_2_	0.3496	0.9066	X_14_	0.4573	0.3335
X_3_	0.3578	0.9092	X_15_	0.0397	0.9870
X_4_	0.2400	0.6268	X_16_	0.0632	0.9966
X_5_	0.6208	0.2241	X_17_	0.2535	0.8707
X_6_	0.1683	0.8122	X_18_	0.1344	0.9279
X_7_	0.1882	0.6816	X_19_	0.2980	0.7416
X_8_	0.7358	0.1038	X_20_	0.3909	0.8995
X_9_	0.1234	0.8777	X_21_	0.0316	0.9643
X_10_	0.1311	0.9468	X_22_	0.3877	0.5661
X_11_	0.3443	0.7354	X_23_	0.4750	0.3018
X_12_	0.3041	0.5848	X_24_	0.2200	0.7100

**Table 5:** Results of interaction between two covariates.

Criterion	Interaction	Interaction factor pairs
q(X_1_ ∩ X_2_) < Min(q(X_1_),q(X_2_)	Nonlinear weakening	No
Min(q(X_1_),q(X_2_)) < q(X_1_X_2_) < Max(q(X_1_)),q(X_2_))	Single-factor nonlinear weakening	No
q(X_1_ ∩ X_2_) > Max(q(X_1_),q(X_2_))	Two-factor enhancement	X_1_ ∩ X_2_, X_1_ ∩ X_3_, etc., a total of 103 pairs
q(X_1_ ∩ X_2_) = q(X_1_) + q(X_2_)	Independence from each other	No
q(X_1_ ∩ X_2_) > q(X_1_) + q(X_2_)	Nonlinear enhancement	X1 ∩ X4, X1 ∩ X6, etc., a total of 173 pairs

q(X_1_ ∩ X_2_) represents the interaction of q(X_1_) and q(X_2_); Min(q(X_1_),q(X_2_)) and Max(q(X_1_)),q(X_2_)) mean the minimum and maximum values among q(X_1_) and q(X_2_), respectively; q(X_1_) + q(X_2_) is the sum of q(X_1_) and q(X_2_)^25^.

As shown in Table 4, among all the influencing factors, the proportion of the rural population with high school or secondary specialized school and above (X_8_), urban construction land area (X_5_), and secondary and tertiary industrial GDP (X_1_) had the largest q value, with 0.7358, 0.6208, and 0.5866, respectively. It shows that these were the three most important influencing factors in all the independent variables determining the spatial pattern of URI in Shandong Province. Its interpretation power of URI space pattern was 73.58%, 62.08%, and 58.66%, respectively.

Table 5 was collated by Interaction detector matrix, which was calculated by Geodetector software. We could conclude from Table 5 that interaction between the two independent variables and the spatial pattern of URI was greater than each independent variable act alone among all the influencing factors (independent variables) of URI in Shandong province. Of these, the interactions of 103 pairs of independent variables, for example, X_1_ ∩ X_2_, X_1_ ∩ X_3_, etc., were two-factor enhancement, while interactions of 173 pairs of independent variables, such as X_1_ ∩ X_4_, X_1_ ∩ X_6_, etc., were nonlinear enhancement with significant interaction effects, showing 1 + 1 > 2 interaction effect. It indicated that the level of URI of each city in Shandong province resulted from the positively comprehensive function of the influencing factors.

#### Spatial heterogeneity of factors influencing URI in Shandong province

**Figure 7:**
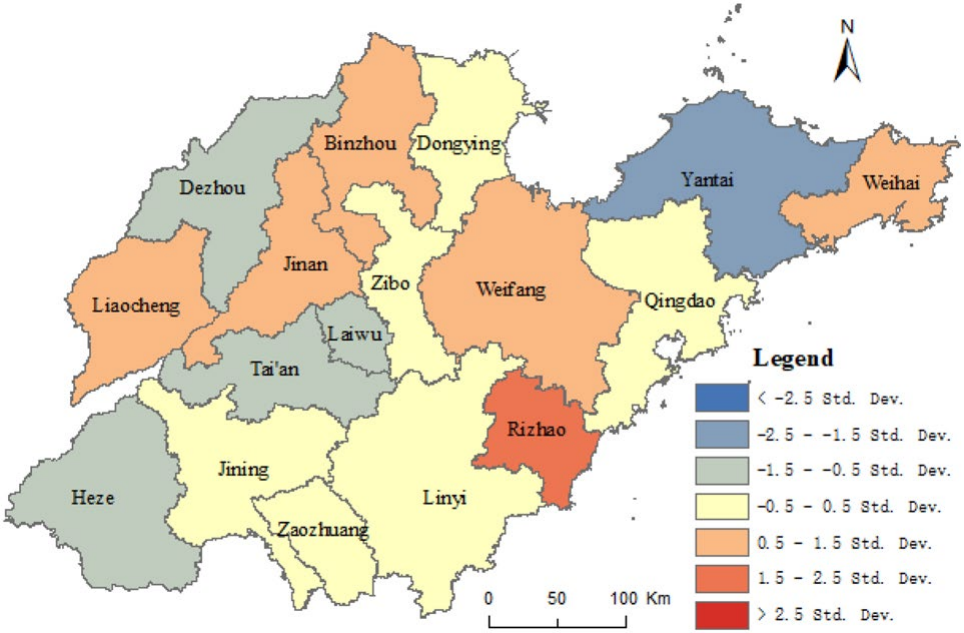
The results of standard error. This figure was created with ArcGIS 10.5 (URL: http://www.esri.com/).

**Figure 8:**
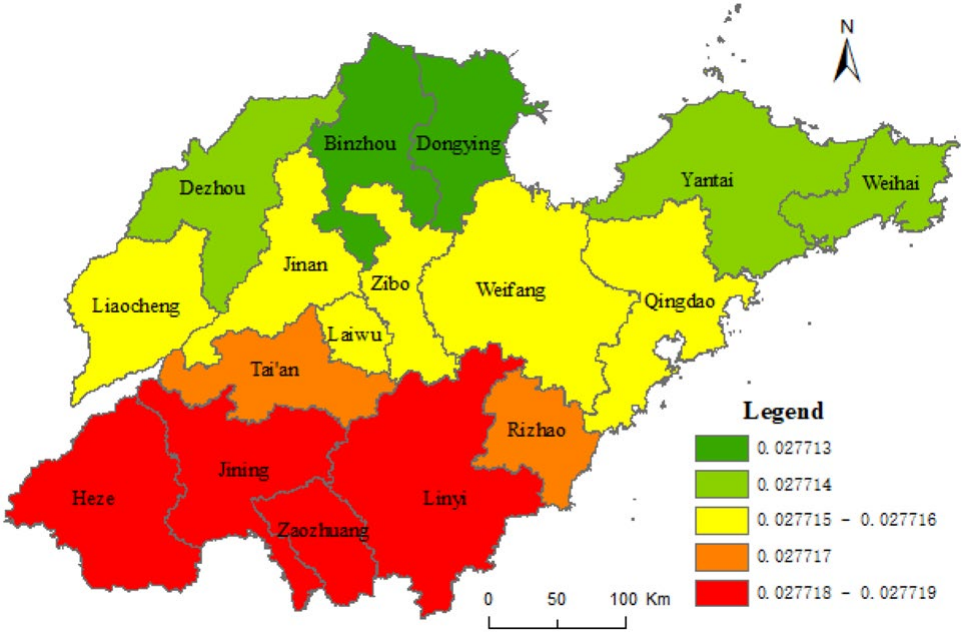
Coefficient of X_8_. This figure was created with ArcGIS 10.5 (URL: http://www.esri.com/).

**Figure 9:**
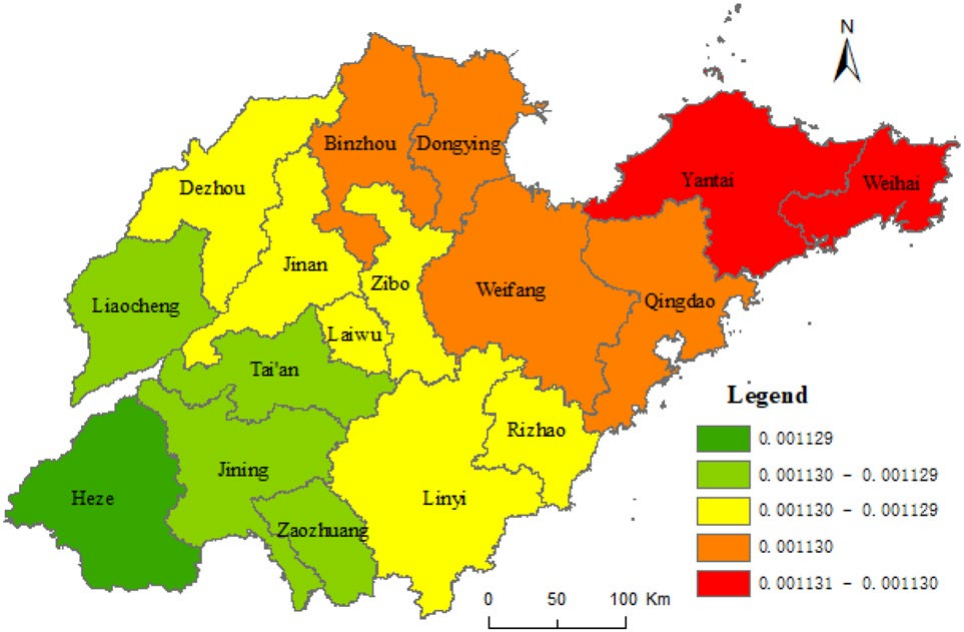
Coefficient of X_5_. This figure was created with ArcGIS 10.5 (URL: http://www.esri.com/).

**Figure 10:**
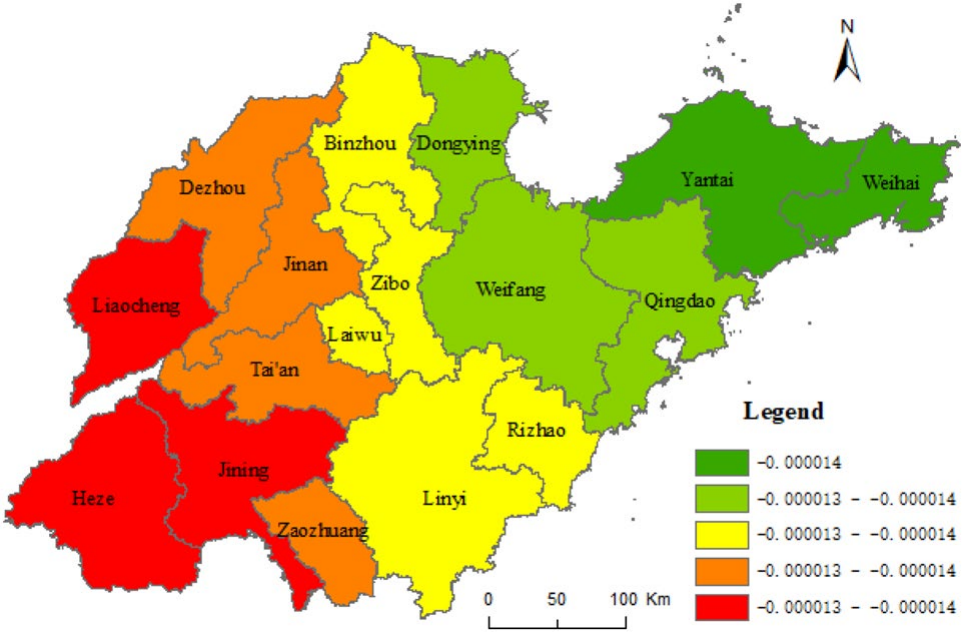
Coefficient of X_1_. This figure was created with ArcGIS 10.5 (URL: http://www.esri.com/).

Based on the results of the geographical survey of the factors affecting the URI, taking the composite index of URI level of Shandong province as dependent variables, and taking the proportion of the countryside population with high school or secondary specialized school and above, urban construction land area, and secondary and tertiary industrial GDP as independent variables, the geographical weighted regression analysis was carried out by the spatial statistical toolbox of ArcGIS 10.5, with the geographically weighted regression analysis method (Formula 8). Figure 7 shows the standard error result of the geographically weighted regression analysis and the coefficients X_8_, X_5_, and X_1_ in Figs. 8, 9, 10, respectively.

Figure 7 shows some spatial difference in the standard error of geographically weighted regression prediction results of urban and rural integration levels in Shandong province. The projected result was lower than the actual standard deviation 1.5 times, while Rizhao was 1.5 times higher. The standard errors of prediction results in other cities were less than 1.5 times the standard difference. Among them, the standard deviation of Qingdao, Dongying, Zibo, Linyi, Zaozhuang, and Jining was less than half a standard deviation. As shown in Figs. 8, 9, 10, the proportion of the rural population with high school or secondary specialized school or above (X_8_), urban construction land area (X_5_), and secondary and tertiary industrial GDP (X_1_) had obvious spatial differences on the level of URI in Shandong province. Among them, the effect of X_8_ on urban–rural integrated levels decreased from south to north, while the effect of X_5_ on the urban–rural integrated level decreased from northeast to southwest. Effects of X_1_ on the urban–rural integrated level decreased from west to east.

## Conclusion and recommendation

Based on the previous result, we can draw the following conclusions: (1) The cities of the highest and higher level of URI all were located along the Qingdao–Jinan railway and the coast, while the cities of the lowest and lower level were in Southwest Shandong and Northwestern Shandong. The spatial pattern of the urban–rural integrated level was consistent with the development level of regional economy. Central cities of the province field, group cities, and economically developed cities were more conducive to drive URI. (2) Spatial agglomeration characteristics of urban–rural integrated level in Shandong were not significant, and the local spatial autocorrelation of 14 of 17 cities was not significant. Yantai and Weifang were High-High cluster areas, while Jining was a Low–Low cluster area. Regions with High-Low outliers and Low–High outliers were not detected. (3) The main factors affecting the level of URI included rural self-development level, the influence of central cities, and connection between cities and countryside. The proportion of rural population with high school or secondary specialized school or above, urban construction land area, and secondary and tertiary industrial GDP were critical factors, influencing the urban–rural integrated level in Shandong. The explanatory power of these three factors to the spatial differentiation of urban–rural integration is 73.58%, 62.08% and 58.66% respectively. In addition, the freight volume has a great explanatory power on the spatial differentiation of urban–rural integration, which is 47.5%. The interaction of any two factors on the spatial pattern of URI was greater than the result of each factor acting alone among all influencing factors. (4) The effect of the proportion of the countryside population with high school or secondary specialized school and above (X_8_), urban construction land area (X_5_), and secondary and tertiary industrial GDP (X_1_) on the urban–rural integrated level in Shandong presented obvious spatial heterogeneity. Spatial heterogeneity is mainly caused by the differences of economic development foundation, education level, population migration, city size, industrial structure, technical level, human resources and other factors in different regions of Shandong Province.

According to the results, we made the following suggestions: (1) Overall, the cultural quality of the rural population should be promoted positively. The above-mentioned studies show that culture quality of the rural population and the proportion of the labor population of the right age are essential for URI. Therefore, the important measure for promoting urban–rural integrated level is to enhance countryside education, strengthen the policy of attracting talent, and improve the level of cultural knowledge of the rural population. (2) Central cities in driving rural areas should fully play their roles. The influence of the central city is crucial to urban and rural integration. So, administrative regional restrictions should be breakthroughs, promoting effect of important central cities on URI of the province by giving full play, and the effect of the provincial center city, regional central city, and specifically important central cities, such as Qingdao, Jinan, and Yantai on the URI of the province, should be further tapped. In addition, we can use long time series nighttime light data to analyze the dynamic direction of central city expansion and spatial impact, and to identify the advantages and disadvantages of URI development. (3) The urban–rural connection needs further strengthening, with convenience directly influencing the spatial pattern of URI. Therefore, concerning traffic conditions and traffic facilities, there is a great need to strengthen the construction. Meanwhile, urban and rural communication needs to drive a smooth channel of contact for urban and rural integration and strength exchanges and cooperation between regions. The economic development level of the areas along the Qingdao–Jinan railway and coastal cities is relatively high. It is necessary to further strengthen its links with other regions to drive the economic development of the whole province. Due to strong complementarity, apparent spatial differences, and the great space to promote each other and improve together from influencing factors of URI in each city, the level of URI needs to improve in response to actual conditions of a region and at advantages and compensation for the deficiency according to regional differences. (4) It is necessary to further advance the process of urbanization, actively develop the secondary and tertiary industries. The proportion of secondary and tertiary industries in GDP of Shandong Province is 3.3% and 3.2% lower than that of Guangdong Province and Jiangsu Province respectively. We should further improve the proportion of the two industries to power for the promotion of URI.

## Data Availability

The datasets generated during and/or analyzed during the current study are available from the corresponding author on reasonable request.

## References

[1] Huang Y, Cai B, Zheng Y (2019). Integration of urban and rural development in the New Era: Present situation problems and countermeasures. Urban Dev. Studies..

[2] Chen KQ, Long HL (2019). Impacts of land market on urban-rural integrated development in China. J. Nat. Resour..

[3] Chen C, LeGates R, Zhao M, Fang C (2018). The changing rural-urban divide in China's megacities. Cities.

[4] Yang Y, Bao W, Wang Y, Liu Y (2021). Measurement of urban-rural integration level and its spatial differentiation in China in the new century. Habitat Int..

[5] Bjørkhaug H, Knickel K (2018). Rethinking the links between farm modernisation, rural development and resilience. J. Rural. Stud..

[6] Plummer P, Tonts M, Argent N (2018). Sustainable rural economies, evolutionary dynamics and regional policy. Appl. Geogr..

[7] Liu Y, Li Y (2017). Revitalize the world’s countryside. Nature.

[8] Liu Y (2018). Research on the urban-rural integration and rural revitalization in the new era in China. Acta Geogr. Sin..

[9] Wang F, Sun H, Sun F (2009). Spatial Differences of Urban-Rural Development Coordination in Shandong Province. Scientia Geographica Sini-ca..

[10] Xu K, Fang Y (2019). Spatial differentiation and type identification of rural territorial multi-functions in Liaoning Province. Geogr. Res..

[11] Liu Y, Zhou Y, Liu J (2016). Regional differentiation characteristics of rural poverty and targeted poverty alleviation strategy in China. Bull. Chin. Acad. Sci..

[12] Wang Y, Liu Y, Yan B, Li Y (2016). Spatial patterns and influencing factors of urban-rural coordinated development in China. Scientia Geographica Sinica..

[13] Long, H., Li, Y., Liu, Y., Woods, M., Zou JJLup Accelerated restructuring in rural China fueled by ‘increasing vs. decreasing balance’land-use policy for dealing with hollowed villages. 29(1):11–22 (2012).

[14] Zhou C, Wu F, Zhang J (2017). The occult metastases and its measurement of urban and rural factor income in China. Stat. Res..

[15] Lyu W, Xu H (2015). Land finance, urban preference and the urban-rural income gap in China. Finance Trade Econ..

[16] Liu Y, Yan B, Wang Y (2016). Urban-rural development problems and transformation countermeasures in the new period in China. Econ. Geogr..

[17] Long H, Tu S (2018). Theoretical thinking of rural restructuring. Prog. Geogr..

[18] Long H, Tu S (2017). Rural restructuring: Theory, approach and research prospect. Acta Geogr. Sin..

[19] Fu, W. Rural industry and its social foundation in the process of urban-rural integration development—Taking the processing of imported materials in remote villages of L City, Zhejiang Province as an example. *Chin. Soc. Sci.*, **41**(6), 71–90, 205–206 (2018).

[20] Long H, Zhang Y, Tu S (2018). Land consolidation and rural vitalization. Acta Geogr. Sin..

[21] Liao Z, Wang L, Yang W (2019). Economic agglomeration and development of regional urban-rural integration—an empirical analysis based on spatial econometric model. Soft Sci..

[22] Li W (2015). Will segmentation of urban-rural move towards Integration?—Based on theoretical and empirical analysis of spatial economics. Finance Econ..

[23] Qiao W, Ge D, Gao J, Lu C, Huang L (2019). Detecting the pathways towards rural vitalization from the perspective of territorial functions in Jiangsu Province. Geogr. Res..

[24] Liu R, Hu J, Wang X (2019). Spatial-temporal features and influencing factors of urban-rural integration development in Northwest China. J. Lanzhou Univ. (Soc. Sci.)..

[25] Xie S, Zhou F, Wu T, Fu C (2020). Evaluation and spatial pattern evolution of urban and rural integrated development in the Yangtze River Delta. Urban Dev. Studies..

[26] Yang F, Yang Y, Lin Y (2020). Measurement of urban-rural integration development and analysis of regional differences. Prices Monthly..

[27] Zhang H, He R, Li G, Wang J (2020). spatiotemporal evolution of coupling coordination degree of urban-rural integration system in metropolitan area and its influencing factors: Taking the capital region as an example. Econ. Geogr..

[28] Zhang X, Qiu F, Zhu C (2020). Evolution of urban-rural integration in Huaihai Economic Zone from the perspective of spatio-temporal interaction. J. Nat. Resour..

[29] Zhou J, Zou W, Qin F (2020). Review of urban-rural multi-dimensional integration and influencing factors in China based on the concept of equivalence. Geogr. Res..

[30] Zhang H, He R, Li L, Li G (2021). Spatio-temporal differentiation of urban-rural integration level and rural revitalization path in the Capital Region. J. Nat. Resour..

[31] Shan B, Liu Y, Wang J (2020). Spatial pattern and influencing factors of urban and rural differences in Shandong Province. Res. Dev..

[32] Wu Y, Li H (2020). Spatial change and correlations of desakota regions in a metropolitan area using NPP/VIIRS nighttime light data: A case study of Wuhan City. Prog. Geogr..

[33] Zhang Z, Shan B, Wang J, Liu Y, Lin Q (2020). The spatial-temporal pattern of urban expansion in Shandong Province based on night light data. J. Shandong Jianzhu Univ..

[34] Shandong Provincial Bureau of Statistics (2021). Statistcal Yearbook of Shandong.

[35] Xu J (2006). Quantitative geography (2nd edition).

[36] Deng Y, Cui Y, Lu W, Zhao M (2021). Research on spatial heterogeneity and influencing factors of china’s low-carbon agriculture development level at city scale: inspection from planting industry. Resour. Environ. Yangtze Basin..

[37] Wang J, Liao Y, Liu X (2019). Spatial data analysis Tutorial (2nd edition).

[38] Wang J, Xu C (2017). Geodetector: Principle and prospective. Acta Geogr. Sin..

[39] Huang X (2014). Income gap, deepening of human capital in the rural areas and the integration of city and the countryside. Economist..

